# Theoretical Upper and Lower Limits for Normalized Bandwidth of Digital Phase-Locked Loop in GNSS Receivers

**DOI:** 10.3390/s23135887

**Published:** 2023-06-25

**Authors:** Young-Jin Song, Thomas Pany, Jong-Hoon Won

**Affiliations:** 1 Autonomous Navigation Laboratory, Department of Electrical and Computer Engineering, Inha University, Incheon 22212, Republic of Korea; yj.song@inha.edu; 2 Institute of Space Technology and Space Applications, University of Federal Armed Forces Munich, 85577 Neubiberg, Germany; thomas.pany@unibw.de; 3 Department of Electrical Engineering, Inha University, Incheon 22212, Republic of Korea

**Keywords:** coherent integration time, global navigation satellite system (GNSS) receiver, loop noise bandwidth, normalized bandwidth, phase-locked loop (PLL), signal tracking loop, stability analysis

## Abstract

Determining the loop noise bandwidth and the coherent integration time is essential and important for the design of a reliable digital phase-locked loop (DPLL) in global navigation satellite system (GNSS) receivers. In general, designers set such parameters approximately by utilizing the well-known fact that the DPLL is stable if the normalized bandwidth, which is the product of the integration time and the noise bandwidth, is much less than one. However, actual limit points are not fixed at exactly one, and they vary with the loop filter order and implementation method. Furthermore, a lower limit on the normalized bandwidth may exist. This paper presents theoretical upper and lower limits for the normalized bandwidth of DPLL in GNSS receivers. The upper limit was obtained by examining the stability of DPLL with a special emphasis on the digital integration methods. The stability was investigated in terms of *z*-plane root loci with and without the consideration of the computational delay, which is a delay induced by the calculation of the discriminator and the loop filter. The lower limit was analyzed using the DPLL measurement error composed of the thermal noise, oscillator phase noise, and dynamic stress error. By utilizing the carrier-to-noise density ratio threshold which indicates the crossing point between the measurement error and the corresponding threshold, the lower limit of the normalized bandwidth is obtained.

## 1. Introduction

Several traditional signal tracking loop architectures, such as phase-locked loops (PLLs) for carrier phase tracking, are widely used as engineering standards in modern digital global navigation satellite system (GNSS) receivers. These architectures ensure optimal carrier phase tracking loop performance by minimizing phase noise jitter at a steady state while constraining the transient error for specified changes in the signal phase at a fixed amount [1,2].

One solution is offered by the variational method for optimization using a Lagrangian multiplier. An optimum solution for PLLs in the continuous-time domain is given by a function of the loop noise bandwidth, which can be interpreted as normal filter coefficients such as decay ratio, damping ratio, and natural frequency [1]. The same method (minimization method) can be applied in the discrete-time domain by directly employing the *z*-transform [3]. Finally, the filter order *n*, the noise bandwidth *B*, and the coherent integration time *T* (i.e., the loop update interval), which determine the number of integrators and the multiplier coefficients of the digital PLL (DPLL) and its response for different signal dynamics and noise statistics, should be chosen to achieve the design for specific user requirements [2]. The optimal solution for the design parameters varies with the expected environmental conditions of the receiver. For example, dynamic users such as receivers within an aircraft and a missile are benefit from a large *B* and small *T* because of the large gain (to alleviate the dynamic stress) and faster update rate. On the contrary, in the case of the static but weak signal conditions such as indoor users can employ the small *B* and large *T* to raise the post-correlation signal-to-noise ratio while suppressing the noise effect.

A software implementation of this based on a simple numerical integrator for the PLL of a pseudo-noise spread spectrum receiver to accommodate a given set of dynamics specifications was previously presented [4]. A systematic survey of the theoretical and experimental work on this topic for the period between 1960 and 1980 can be found in [5].

Typically, longer *T* in correlators, which are equivalent to longer loop update intervals in loop filters, provide the additional processing gain required for the detection and processing of signals in harsh environments, specifically weak indoor signals and integrated navigation systems [6]. The benefits of the longer *T* using the aiding technique are multipath mitigation in the Doppler domain, cross-correlation protection by data wipe-off, and reduced squaring loss. The use of ancillary sensors with relevant processing facilities was proposed to extend the *T* to several seconds by providing better-matched replica signals [7]. However, there is no further processing gain beyond a certain *T* because of the spatial and temporal decorrelation of the received signal, the residual instability of the receiver oscillator, and data bit transitions, all of which result in a narrowing of the bandwidth [8]. The limits on the *T* due to the spatial decorrelation of multipath signals were derived and experimentally verified using a generalized multipath scattering model [6]. The results showed that the processing gain obtained from longer *T* saturates after the antenna has moved a certain distance, which is typical for indoor propagation.

In addition, one of the dominant error sources in PLLs is dynamic stress error, which is a barrier to further reducing the loop bandwidth *B*. In particular, removing the dynamic stress for a moving platform receiver using external aiding information would be a logical first step towards decreasing the *B* and enhancing the tracking sensitivity in weak signal environments. Therefore, narrower bandwidths are known to be a good solution in weak signal processing because they suppress noise effects. However, both longer *T* and narrower *B* are less robust to signal dynamic stress.

Investigation of the trade-off between the sensitivity and the loop update interval to choose an optimal *T* was performed for a signal tracking Kalman filter with a special emphasis on the discrete nature of the loop, where the carrier phase measurements were asynchronous with the loop update [9].

Furthermore, designing a DPLL relies heavily on transforming a continuous-time system into a discrete-time system, performed by Laplace to a *z*-domain mapping. However, if the loop update interval is large relative to the most significant time constant of the system, the discrete-time system may not be an accurate representation of the corresponding continuous-time system. That is, the larger loop update interval makes the system less stable, and the updated output no longer gives an accurate measure of the output [10].

This means that there is another type of factor, similar to transforming continuous-time systems into discrete data systems. The DPLL will be equivalent to its analog counterpart only if the product *BT* (the so-called normalized loop bandwidth) is close to zero [5,11]. For sufficiently small values of *BT* (e.g., *BT* < 0.1), this can provide an adequate starting point for the analysis and design of DPLLs. However, as *BT* increases, the deviation of the equivalent loop noise bandwidth and the closed-loop pole locations of the designed DPLL from the desired ones in the continuous-time domain eventually leads to the instability of the loop. Therefore, both the limit on *T* and *B* should be considered together [11,12].

Another method (controlled-root method) was proposed to derive the loop filter constants from the loop roots based on the direct physical meaning of the loop noise bandwidth and the root-specific decay rate or damping. In this method, the deviation of discrete-time systems from continuous-time systems for large values of *BT* is avoided. However, the maximum achievable *BT* for a stable loop is limited to 0.4 for a rate-only feedback-type numerically controlled oscillator (NCO) [11,12]. Note that this condition is satisfied for most communication systems because their *BT* value remains within this range. However, for some new applications in GNSSs, such as weak signal tracking, *T* values larger than one navigation data bit duration (e.g., 20 ms) are preferred for high sensitivity after the navigation data bit transition is solved correctly. Moreover, the use of pilot signal components in new GNSSs provides a chance to use the longer *T*, which will inevitably require larger *BT* values.

In this study, although two of the DPLL design methods (minimization, controlled-root) are introduced above, another DPLL design approach, designing the filter in the analog domain and then transforming it to the digital domain by using three types of digital transform methods (step-invariant, impulse-invariant, and bilinear), is used. As it is simpler than designing directly in the digital domain or applying controlled-root formulation, it is still widely employed as an engineering standard in many GNSS receiver designs, especially for high sensitivity. In addition, the computational delay (also called as transport lag), a time delay due to the calculation process for the loop update, is assumed not to exist for the first time of the analysis and is considered later.

The paper concentrates exclusively on the theoretical upper and lower limits of *BT*. The approach taken here for the upper limit is to choose a value of *BT* that satisfies the stability condition based on root loci in the *z*-domain. Each digital transform method is analyzed for the utilization of the loop filters and the NCOs. In addition, the lower limits of *BT* are obtained considering the effect of the *BT* on the oscillator quality and dynamic stress of the DPLL. The contribution of this paper is to provide the baseline for the DPLL designers by suggesting the upper and lower limits as requirements.

The remainder of this paper is organized as follows. In Section 2, PLLs are briefly reviewed with a special emphasis on each digital transformation method for the loop filter and the NCO, considering the effect of the computational delay on the transfer function. Section 3 describes the stability problem encountered near the upper limit of *BT* values for different digital transform methods. The stability of the different digital transformation methods is tested in terms of root loci, *BT* margins, Bode plots, and step responses, with and without the assumption of the computational delay. Section 4 derives the lower limits of *BT* for a few typical scenarios in consideration of the oscillator quality and the dynamic stress of the receiver. In Section 5, the stability issue is numerically tested using a GNSS software receiver and the paper is concluded in Section 6.

## 2. Review of Phase-Locked Loops

This section presents a review of the results widely used in PLL design. Specifically, the mathematical models for PLLs are reviewed in the continuous-time and discrete-time domains, with a special emphasis on each digital integration method implementing the loop filter and the NCO.

### 2.1. Continuous Model

**Figure 1:**
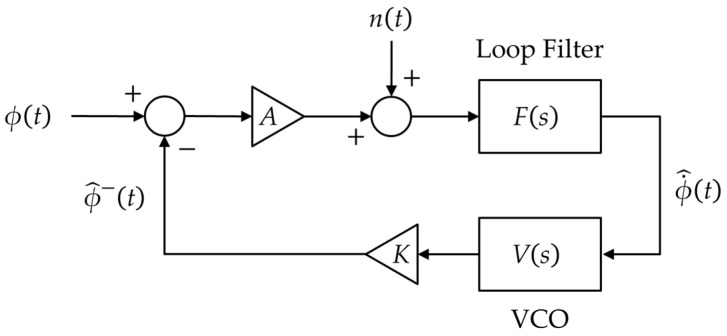
Linearized phase-locked loop (PLL) model in the continuous-time domain.

Using the assumptions that the phase error is small enough and that the input noise is uncorrelated with the incoming waves, the linearized model for a PLL in the continuous-time domain is presented as shown in Figure 1. The device is composed of a phase detector (discriminator), a loop filter *F*(*s*), and a voltage-controlled oscillator (VCO) *V*(*s*) with a discriminator gain *A* and VCO gain *K*, where *s* = *σ* + *jω* denotes the Laplace operator. In the figure, $\phi \left(t\right)$ symbolizes the measured input phase at time *t*, $\hat{\dot{\phi }}\left(t\right)$ and ${\hat{\phi }}^{-}\left(t\right)$ represent the estimates of the phase rate and phase, respectively, and *n*(*t*) denotes the noise.

The purpose of the loop filter is to reduce the influence of the noise to produce an accurate estimate of the original signal. First-/second-/third-order loop filters (selected depending on user requirements) are widely used in GNSS receivers. The loop filter should be designed to satisfy the filter design criteria, namely minimizing VCO phase noise jitter due to noise effects (i.e., minimization of the root-mean-square noise error), and maintaining the transient error in the VCO phase caused by specified changes in the signal phase (i.e., the minimization of the transient error). These criteria are effectively met using the variational method for optimization via the Lagrangian multiplier method for continuous-time PLLs [1] and discrete-time PLLs [3].

The VCO can be uniquely specified using a simple analog integrator represented by 1/*s* (the Laplace transform of an integrator in the continuous-time domain):

(1) $$V\left(s\right)=1/s$$

The closed-loop transfer function of the continuous-time PLL *H*(*s*) is given by:

(2) $$H\left(s\right)=\frac{AKV\left(s\right)F\left(s\right)}{1+AKV\left(s\right)F\left(s\right)}$$

Typically, the feedback loop gain *AK* is assumed to be 1 (i.e., unity gain). Then, the transfer function in (2) can be rewritten as:

(3) $$H\left(s\right)=\frac{V\left(s\right)F\left(s\right)}{1+V\left(s\right)F\left(s\right)}=\frac{F\left(s\right)}{s+F\left(s\right)}$$

Table 1 presents a summary of first-/second-/third-order tracking loops and their characteristics. The filter order and the loop filter natural radian frequency *ω*_0_ (which is obtained from *B*) are determined in the design stage depending on the user requirements. *a_i_* and *b_i_* represent the filter coefficients of the *i*-th-order loop filters [2].

### 2.2. Discrete Model

**Figure 2:**
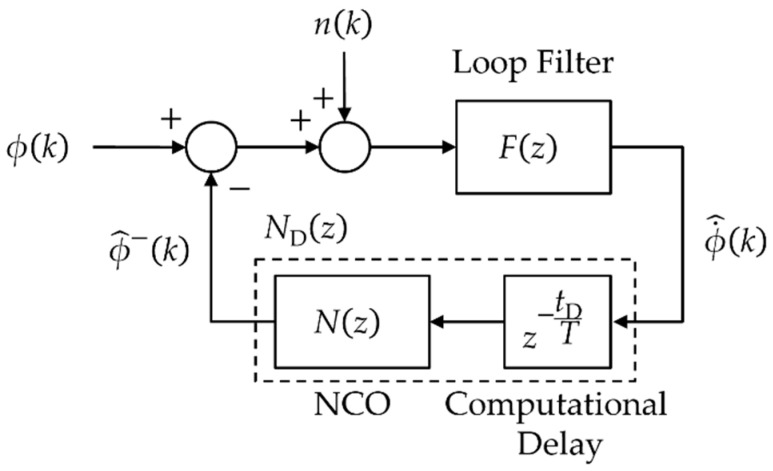
Linearized digital PLL (DPLL) model in the discrete-time domain (assuming unity gain; *AK* = 1).

A block diagram for a DPLL with unity gain is shown in Figure 2. In the figure, *F*(*z*) represents the transfer function of an arbitrary loop filter, and *N*(*z*) is the transfer function of the NCO, where both are digital equivalents of *F*(*s*) and *V*(*s*), respectively, and can be affected by the characteristics of the particular digital implementation of the analog integrator. Additionally, the computational delay is inserted between the loop filter and the NCO to model the effect of the inevitable delay due to the calculation processes of the discriminator and loop filter. The ideal case that does not have such a delay can be represented by substituting *t*_D_ = 0. Finally, the computational delay is combined with *N*(*z*) to form the delay-included NCO transfer function *N*_D_(*z*) later.

Without considering the computational delay, the closed-loop transfer function of the DPLL *H*(*z*) is given by:

(4) $$H\left(z\right)=\frac{N\left(z\right)F\left(z\right)}{1+N\left(z\right)F\left(z\right)}$$

which can be rewritten in polynomial form as:

(5) $$H\left(z\right)=\frac{\beta_{m}z^{m}+\beta_{m-1}z^{m-1}+\cdots +\beta_{0}}{\alpha_{n}z^{n}+\alpha_{n-1}z^{n-1}+\cdots +\alpha_{0}}=\frac{\sum_{j=0}^{m}\beta_{j}z^{j}}{\sum_{i=0}^{n}\alpha_{i}z^{i}}$$

where *α_i_* and *β_j_* are filter coefficients to the *n*-th order polynomial in the denominator and the *m*-th polynomial in the numerator, respectively, and *m* and *n* are the number of zeros and poles, respectively (*n* ≥ *m*; *n* = the order of the loop).

There are three primary categories of methods for obtaining a discrete equivalent of a continuous transfer function because of the discrete nature of the system, as described in the following sections [13].

#### 2.2.1. Hold Equivalent

The hold equivalence method is to design a discrete system with an input consisting of samples of input signals, which has an output that approximates the output of the continuous-time transfer function whose input is the input signals in the continuous-time domain. This design is accomplished by acquiring a sequence of samples and extrapolating or holding them to produce a continuous signal. Depending on the order of the holding approximation, there are several variations in this method, such as zero-order holding and first-order holding. However, this method is only used to model a continuous system, because it relies on the time response of the system being unable to faithfully reproduce the frequency response, whereas filter design of the loop filter used in this work is based on their frequency response characteristics.

#### 2.2.2. Pole-Zero Mapping

The idea behind the pole-zero mapping method is to use a set of heuristic rules to locate the poles and zeros using the map *z* = *e^sT^*. Then, the gain of a *z*-transform is set to describe a discrete equivalent transfer function that approximates the given transfer function in the *s*-plane.

#### 2.2.3. Numerical Integration

To obtain an equivalent discrete model of a PLL that retains the frequency response of the original continuous-time PLL, designers typically apply a numerical integration method (also known as the *z*-transform method). The fundamental concept of this method is to represent the given continuous-time transfer function as a differential equation and to derive a difference equation by replacing *s* in the continuous transfer function with a function of *z*.

Table 2 summarizes the three types of numerical integration methods that are of interest in this paper, including their *z*-domain and discrete-time domain expressions, in addition to some differently used terminology in different fields. These methods are step-invariant (SI), impulse-invariant (II), and bilinear (BL) rules. A detailed digital implementation of these methods for first-/second-/third-order PLLs is presented in the Appendix A.

Given a continuous-time transfer function *H*(*s*), a discrete equivalent *H*(*z*) can easily be found in these methods by substituting *s* with its counterpart equation for *z*, which can itself be derived from the equations in Table 2. Therefore, each of the approximations in Table 2 can be viewed as a mapping function from the *s*-plane to the *z*-plane. To make the system stable in graphical interpretation, the region of the stable poles in the *s*-plane (i.e., the left half of the *s*-plane) should be mapped onto the inside of the unit circle in the *z*-plane [13].

Table 3 lists the resulting *α_i_* and *β_j_* in (5) for the first-/second-/third-order DPLLs for various combinations of digital representations of the integrators in the loop filter *F*(*z*) and NCO *N*(*z*). Note that all the systems are a function of *ω*_0_*T*, such that *BT* determines the overall filter response.

### 2.3. Computational Delay

The previous section derived the coefficients of the closed-loop transfer functions in the discrete domain without considering the computational delay. The computational delay or transport lag is a time delay between the end of the integration process and the new NCO input (i.e., phase rate estimate), which is incurred by the calculation process at the discriminator and the loop filter. The computational delay *t*_D_ exists in hardware and real-time software receivers without an appropriate buffering capability to eliminate this delay and should be considered in the stability analysis [14].

The computational delay can be modeled as an additional delay unit for the amount of *t*_D_ located before the NCO [14], which results in the modification of the NCO transfer function *N*(*z*) as:

(6) $$N_{D}\left(z\right)=z^{-\frac{t_{D}}{T}}N\left(z\right)$$

where the subscript D means the computational delay included. Utilizing the modified NCO transfer function *N*_D_(*z*), the closed-loop transfer function *H*(*z*) of (4) is modified to contain the computational delay as follows:

(7) $$H_{D}\left(z\right)=\frac{z^{-\frac{t_{D}}{T}}N\left(z\right)F\left(z\right)}{1+z^{-\frac{t_{D}}{T}}N\left(z\right)F\left(z\right)}=\frac{N_{D}\left(z\right)F\left(z\right)}{1+N_{D}\left(z\right)F\left(z\right)}$$

By assuming that the new NCO input is obtained from the integration results at the previous epoch of the loop filter, as a typical example, the *t*_D_ becomes *T*. Then, the *N*_D_(*z*) contains unit delay as a computational delay as follows:

(8) $$N_{D}\left(z\right)=z^{-1}N\left(z\right)$$

and is used for the stability analysis of the DPLLs in case of computational delay are considered.

## 3. Stability Analysis for Upper Limit

In practice, DPLLs are a type of sampled-data loop that is never unconditionally stable; high gain loops always result in instability because of the inherent computational delay, which constitutes a major potential drawback of this type of system. Moreover, a stability problem occurs for DPLLs when the loop bandwidth is insufficiently small relative to the loop update interval. Therefore, the upper and lower limits on the value of *BT* that ensure the stability margin and the gain margin of the DPLLs should be determined. The upper limits for DPLLs are obtained from the stability analysis in this Section.

### 3.1. Root Loci

As is well known in control theory, the stability of linear time-invariant systems in the continuous-time domain can be determined by determining the location of the roots of the characteristic equation of the system. For bounded-input–bounded-output stability, the roots of the characteristic equation (i.e., the poles of the system) must all lie in the left half of the *s*-plane. Therefore, the stability of the system can be determined by checking the root locus diagram, which is a pictorial representation of the poles of the closed-loop transfer function as a function of the loop gain.

The root locus diagrams for discrete-data systems are constructed in the *z*-plane using essentially the same properties as those of the continuous-data systems in the *s*-plane, except that the relationship between the root locations and stability must be made concerning the unit circle |*z*| = 1 in the *z*-plane [10]. Here, the loci of roots are defined when only *BT* varies instead of the loop gain *K* (unity gain is assumed for the discriminator gain *A*) to test the stability of a DPLL as a function of *BT*. The closed-loop transfer function of the DPLL in (7) is then rewritten as:

(9) $$H_{D}\left(z\right)=\frac{\left(z-z_{m}\right)\left(z-z_{m-1}\right)\cdots \cdots \left(z-z_{0}\right)}{\left(z-p_{n}\right)\left(z-p_{n-1}\right)\cdots \cdots \left(z-p_{0}\right)}=\frac{\prod_{j=0}^{m}\left(z-z_{j}\right)}{\prod_{i=0}^{n}\left(z-p_{i}\right)}$$

where *p_i_* and *z_j_* represent the poles and zeroes of the system, respectively.

It is obvious that as *B* increases, the roots of the closed-loop transfer functions in the *s*-domain deviate from the origin, but still stay in the left half of the *s*-plane for all values (i.e., the system is unconditionally stable). However, for the second- or higher-order systems, such pole deviation causes oscillation of their step responses.

**Figure 3:**
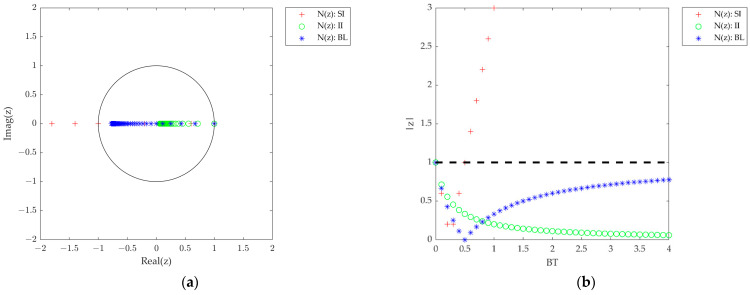

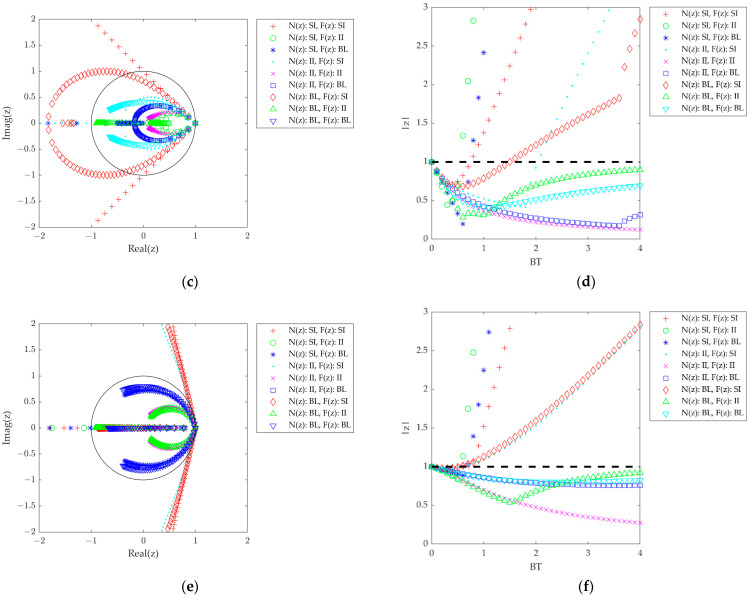
Root location of the DPLL closed-loop transfer functions with three different integration methods for *N*(*z*) and *F*(*z*) (*t*_D_ = 0). (**a**) First-order root loci. (**b**) First-order root magnitude. (**c**) Second-order root loci. (**d**) Second-order root magnitude. (**e**) Third-order root loci. (**f**) Third-order root magnitude.

The left-side figures of Figure 3 illustrate the same effect in the *z*-plane for the first-/second-/third-order closed loops as described above but with different numerical integration methods for *N*(*z*) and *F*(*z*), and with the assumption of the zero computational delays. The trajectory for all cases begins at *z* = 1 when *BT* = 0 and then moves to the inside or outside of the unit circle as *BT* increases. The presence of roots at |*z*| = 1 causes the step response of the system to oscillate with a constant amplitude, and the system becomes marginally stable. Thus, the DPLL will be unstable for |*z*| > 1, even if its counterpart in the continuous-time domain is unconditionally stable. Therefore, the value of *BT* that makes the system marginally stable is the upper limit of available *BT* values for the stable DPLL. The limit is defined as *BT*_osc_ which indicates the *BT* when |*z*| = 1 (i.e., *BT* at the crossing point between the root trajectory and the unit circle) and is the point that makes the system marginally stable, so the step response begins to oscillate at this point.

The right-side figures of Figure 3 show |*z*| for the same case as discussed above with respect to *BT*. As mentioned earlier, |*z*| should be less than 1 for a stable system. For the first-order DPLL in Figure 3b, the root loci for SI are outside the unit circle in the *z*-domain for *BT* > 0.5 (i.e., *BT*_osc_ = 0.5), whereas those for BL and II converge to values on the unit circle (|*z*| = 1) and the origin (|*z*| = 0), respectively, as *BT* increases. A similar phenomenon is observed in the second- and third-order cases. Therefore, three types of DPLL systems are identified in terms of system stability while varying *BT* as follows (see Table 4):

1. Type A—The poles drift outside the unit circle in the *z*-domain as *BT* increases. The system is stable only when *BT* ≤ *BT*_osc_, otherwise it is unstable;
2. Type B—All of the poles are within the unit circle but approach |*z*| = 1 as *BT* increases. The instability of the system increases as *BT* increases;
3. Type C—All of the poles of the system are located inside the unit circle. The system is unconditionally stable, even for large *BT* values.

**Figure 4:**
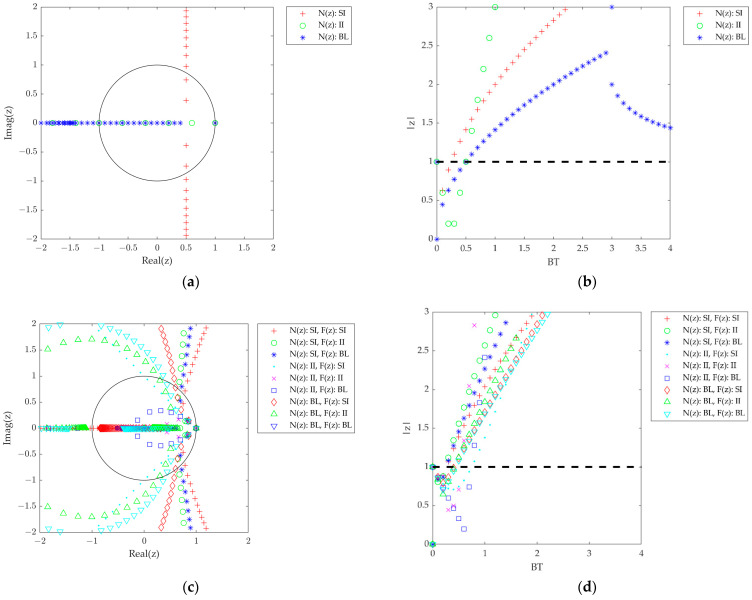

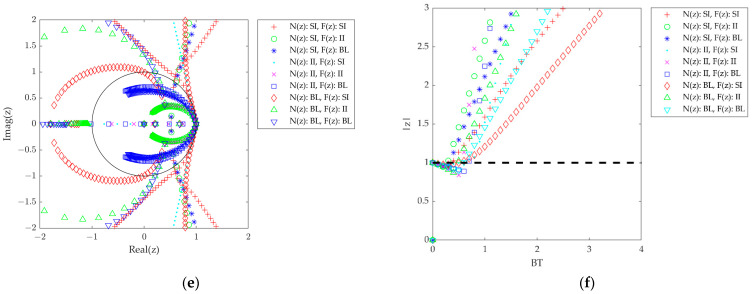
Root location of the DPLL closed-loop transfer functions with three different integration methods for *N*(*z*) and *F*(*z*) (*t*_D_ = *T*). (**a**) First-order root loci. (**b**) First-order root magnitude. (**c**) Second-order root loci. (**d**) Second-order root magnitude. (**e**) Third-order root loci. (**f**) Third-order root magnitude.

Figure 4 presents the same plots as Figure 3, but the unit delay is included in the NCO transfer function as (8) for the assumption of the computational delay (i.e., *t*_D_ = *T* assumed) in this case. Unlike the zero computational delay case, regardless of the used digital integration method, all the root loci magnitude exceed |*z*| = 1 as the *BT* increases. For instance, as can be seen in Figure 4b, the II and BL of the first-order DPLL, which are stable for all *BT*s with zero computational delays, are now stable only at *BT* ≤ 0.51 (i.e., *BT*_osc_ = 0.51) because of the computational delay. Similar results can be observed for the second- and third-order DPLLs. Therefore, it is viewed that the computational delay in the NCO degrades the stability of the DPLLs. Consequently, such DPLLs are conditionally stable.

Table 5 summarizes the stability conditions of the *BT* for different integration methods in the loop filter and the NCO. For the zero computational delays, the II method plays an important role in making the system unconditionally stable (Type C) and the SI method forces the system to be conditionally stable (i.e., Type A). However, as discussed above, if the unit computational delay is included in the stability analysis, all the methods are conditionally stable (i.e., Type A) regardless of the filter order or integration method. Furthermore, all the *BT*_osc_ for *t*_D_ = *T* are located within 0.25 and 0.75 which does not exceed 1, so the results are matched with the well-known rule-of-thumb *BT* threshold (*BT* ≈ 1) for the stable operation of the DPLL.

### 3.2. BT Margin

In system theory, the gain margin is the ratio of the maximum loop gain with stability to the loop gain at the design point and is used to determine the region of stability. Typically, the gain margin is obtained using the ratio of the minimum damping parameter that produces instability to the minimum damping parameter at the design point because the loop gain is proportional to the damping parameter. A similar concept is utilized to determine the region of stability of a system that has a unity gain for different values of *BT*.

Defining the normalized bandwidth margin as the ratio of the maximum *BT* value with stability to the *BT* value at the design point, the margin can be calculated as follows:

(10) $$BTM=\frac{BT_{osc}}{BT}$$

Note that the normalized bandwidth margin is inversely proportional to *BT* and that for the same value of *B* within the stable region (i.e., *BT* < *BT*_osc_), a larger loop update interval makes the system reduce the capacity of the sampled output to accurately measure the true output.

### 3.3. Step and Frequency Responses

**Figure 5:**
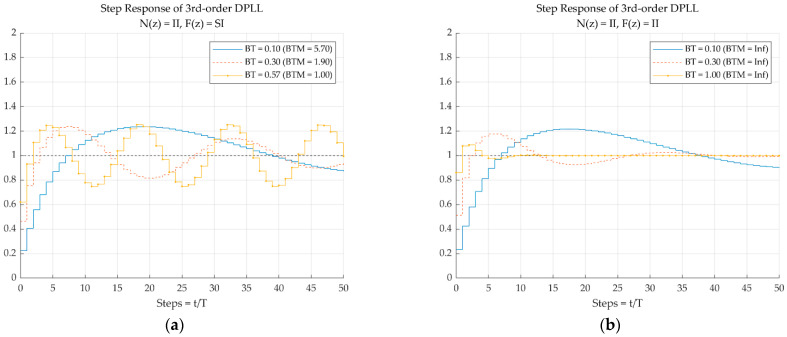
Step responses of the third-order closed-loop transfer function of the DPLL with different numerical integrations for *N*(*z*) and *F*(*z*) for different *BT* (*t*_D_ = 0). (**a**) Type A—*N*(*z*) = II and *F*(*z*) = SI (**b**) Type C—*N*(*z*) = II and *F*(*z*) = II.

Figure 5 shows an example of the step response of the third-order closed loop function of DPLLs with the numerical integration combination of *N*(*z*) = II and *F*(*z*) = SI [Type A in Figure 5a] and II [Type C in Figure 5b] for three *BT* values. Since all the DPLL types are the same when the computational delay is included, zero computational delays are assumed here to observe the behaviors of the different types. The Type A system oscillates rapidly as *BT* increases and becomes unstable when *BT* = 0.57, which is the value of *BT*_osc_ for the II-SI combination when *t*_D_ = 0 (see Table 5), whereas the Type C system remains stable, even for *BT* = 1.

**Figure 6:**
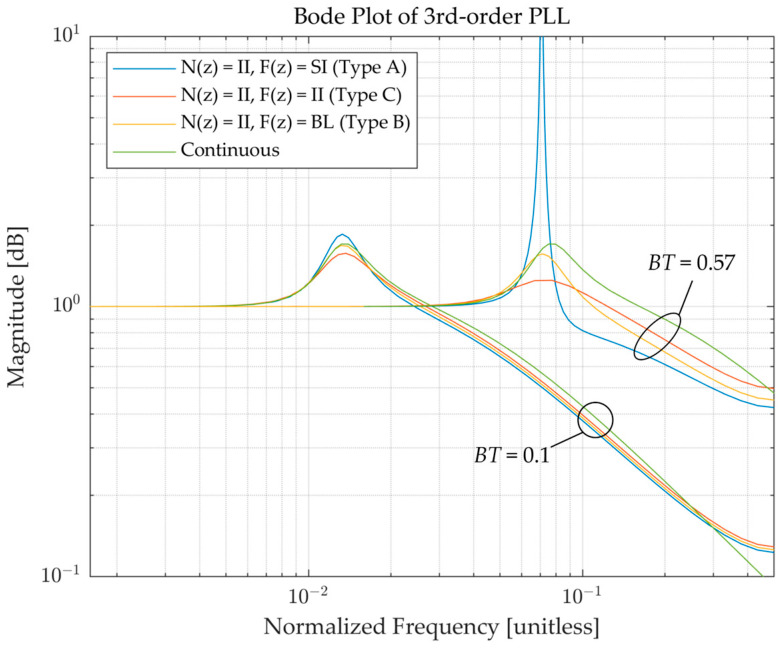
Bode plots of the third-order closed-loop transfer functions of the PLL with different numerical integrations for *N*(*z*) and *F*(*z*) for different *BT* (*t*_D_ = 0).

Figure 6 presents the Bode plots of the same case as discussed above with the addition of *F*(*z*) = BL (Type B) and its continuous-time domain counterpart for small and large *BT*. When *BT* is sufficiently small relative to *BT*_osc_, the frequency responses of the DPLLs accurately represent the system, and little distortion is observed for all numerical integration methods. However, the distortion causes the DPLLs to oscillate at maximum amplitudes at larger *BT* when *BT* approaches the system’s resonant frequency *BT*_osc_. This clarifies the fact that the design parameter for the PLL is the bandwidth of the loop *B*; however, the normalized bandwidth *BT* in DPLL does not reflect the true noise equivalent bandwidth of the loop as *BT* increases, and at the same time, the deviation of the actual bandwidth of the digital system is affected by the type of numerical integration method.

## 4. *BT* Lower Limit

As revealed from the previous section, the *BT* upper limits exist for each numerical integration method for the DPLL, where *BT*s larger than such a limit cannot assure the stable operation of the DPLL. Similarly, lower limits can exist that the *BT*s lower than the limit fails to track the carrier phase successfully. The reason for this is that the gain (i.e., *BT*) of the tracking loop is too small to catch up with the dynamics induced by the receiver dynamic stress and the oscillator phase noise. Therefore, the *BT* lower limits can be drawn by analyzing the DPLL measurement error with its threshold for stable tracking. Here, the third-order DPLL is considered for the sake of simplicity, however, a similar approach can be applied to first-/second-order DPLLs analogously.

### 4.1. Measurement Error

The DPLL measurement error is the standard deviation of the estimated phase error in the DPLL, and the thermal noise contributes a huge portion of it. Nevertheless, the oscillator phase noise and dynamic stress should be taken into account if a low-quality oscillator is used as the clock source, or the receiver faces large dynamics. The oscillator phase noise induced by the vibration is not considered in this work for the simplicity of analysis. The standard deviation of each error source is obtained individually and merged later to form the overall measurement error which can be compared with the threshold.

#### 4.1.1. Thermal Noise

The thermal noise of the Costas PLL which is used for the phase tracking of the data channel (i.e., phase transition existing channel) is modeled as [2]:

(11) $$\sigma_{t}=\frac{180}{\pi }\sqrt{\frac{B}{C/N_{0}}\left(1+\frac{1}{2TC/N_{0}}\right)}\left[deg\right]$$

where *C/N*_0_ is the carrier-to-noise density ratio expressed in a linear scale [Hz].

#### 4.1.2. Allan Deviation Phase Noise

An ideal oscillator is represented as a sinusoidal wave, but a realistic oscillator suffers from phase modulation which has a random characteristic. The phase modulation results in frequency instability which can be described using the Allan deviation. Finally, the phase noise of the third-order DPLL induced by the Allan deviation is given by [15]:

(12) $$\sigma_{A}=\frac{180}{\pi }\sqrt{2\pi^{2}f_{c}^{2}\left(\frac{\pi^{2}h_{-2}}{3\omega_{0}^{3}}+\frac{\pi h_{-1}}{3\sqrt{3}\omega_{0}^{2}}+\frac{h_{0}}{6\omega_{0}}\right)}\;\left[deg\right]$$

where *f*_c_ is the carrier frequency [Hz] and *h*_−2_, *h*_−1_, *h*_0_ are the clock parameters which are determined by the quality of the oscillator. As can be observed from the equation, *σ*_A_ is inversely proportional to the $\omega_{0}$, which is proportional to the *B*. That is, DPLLs with sufficient *B* can effectively suppress the clock jitter, otherwise, the phase tracking performance is determined by the quality of the oscillator. In this study, two types of oscillators are utilized for the phase noise analysis, namely a temperature-compensated crystal oscillator (TCXO) and oven-controlled crystal oscillator (OCXO). Clock parameters are obtained using the model provided by [15], which are listed in Table 6.

#### 4.1.3. Dynamic Stress Error

The DPLL suffers from the dynamic stress error when the receiver-satellite line-of-sight (LOS) range varies rapidly, for example, if the receiver moves fast. The endurable order of the dynamic stress is related to the order of the DPLL as described in Table 1. The dynamic stress error of the DPLL is incurred by the dynamic component that has higher order than the trackable component of the DPLL and can be generally modeled as [2]:

(13) $$\theta_{e}=\frac{d^{n}R/dt^{n}}{\omega_{0}^{n}}\left[deg\right]$$

where *n* is the loop filter order and $d^{n}R/dt^{n}$ is the LOS dynamic stress [deg/s*^n^*] of the *n*-th order DPLL. The typically used second-/third-order DPLLs for the GNSS receiver suffer from the $d^{2}R/dt^{2}$ and $d^{3}R/dt^{3}$, which are the acceleration stress and jerk stress, respectively.

#### 4.1.4. Overall Measurement Error

The overall DPLL measurement error is composed of the thermal noise, oscillator phase noise, and dynamic stress error as follows [2]:

(14) $$\sigma_{DPLL}=\sqrt{\sigma_{t}^{2}+\sigma_{A}^{2}}+\frac{\theta_{e}}{3}\leq 15\;deg$$

and can be calculated by substituting (11) to (13) into (14). The 3*σ*_DPLL_ must not exceed the rule-of-thumb DPLL tracking threshold (i.e., 45 deg), which is obtained by the 1/4 of the pull-in range (i.e., 180 deg) of the Costas PLL discriminator, consequently, *σ*_DPLL_ should be less than 15 deg to not to loss lock [2].

### 4.2. Lower Limit

**Figure 7:**
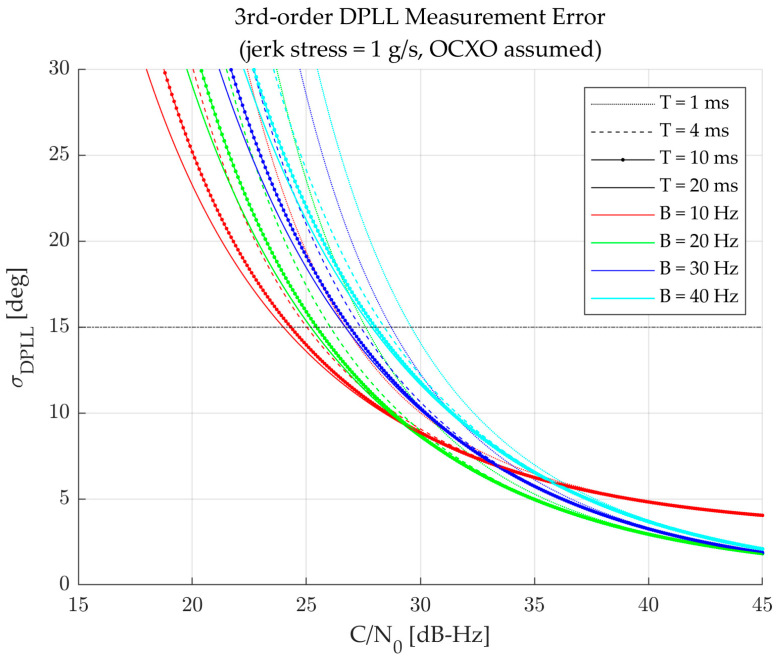
Third-order DPLL measurement error curves for the *C/N*_0_ for various *B* and *T* (jerk stress of 1 g/s and OCXO assumed).

Since the *σ*_t_ in (11) is a function of the *C/N*_0_, the resulting *σ*_DPLL_ in (14) varies with the given *C/N*_0_. Figure 7 illustrates the *σ*_DPLL_ curves for the *C/N*_0_ for various *B* and *T* values, as an example. Obviously, the measurement error increases as the *C/N*_0_ decreases, and eventually crosses the measurement error threshold line (i.e., horizontal line at 15 deg). As the DPLL cannot operate above the threshold line, the *C/N*_0_ at the crossing point indicates the minimum *C/N*_0_ that the specific DPLL can track, which can be defined as a *C/N*_0_ threshold for the DPLL as:

(15) $$C/N_{0,thresh}=\underset{C/N_{0}\in R}{\mathrm{arg min}}\left|\sigma_{DPLL}-15\right|$$

**Figure 8:**
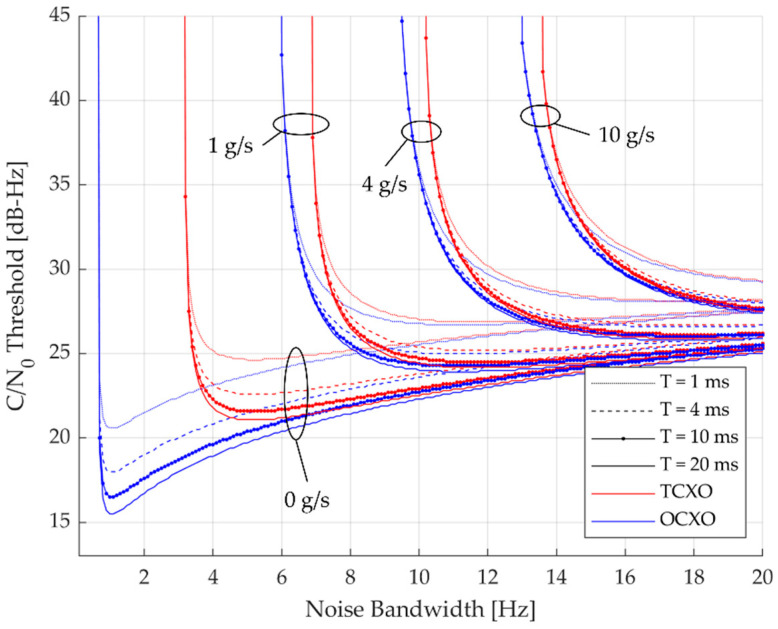
*C/N*_0_ threshold curves for *B* of third-order DPLL using TCXO and OCXO.

The *C/N*_0_ thresholds of third-order DPLL for each measurement error parameter are calculated to form the *C/N*_0_ threshold curves presented in Figure 8. As the *B* becomes narrower, the *C/N*_0_ threshold declines smoothly, and at some point, it rapidly rises. Such vertical lines represent the lower limits of the *B*, which means DPLLs that have *B*s smaller than this limit have insufficient gain to work well in that condition. Note that, as can be observed from the figure, the lower limit is dependent on the *B*, not *BT*, and *T* has a negligible impact on the lower limits. Therefore, the lower limits with respect to the *B* are induced first and the *T* is multiplied later to form the *BT* that is the main interest of this paper. The *BT* lower limit is represented as follows:

(16) $$\begin{array}{ll} BT_{low} & =T\times B_{min}\\ & =T\times \underset{B\in \left(0,\infty \right)}{\mathrm{arg max}}\left|\frac{\partial }{\partial B}\left(C/N_{0,thresh}\right)\right| \end{array}$$

where *B*_min_ is the minimum *B* [Hz] that the DPLL can operate at specific environmental conditions. In the case of no dynamic stress (0 g/s case), the *B* lower limits of TCXO and OCXO deviate from each other because all the phase noise observed as a dynamic component at the receiver is dominated by the oscillator phase noise. Clearly, the *B* limit of the OCXO is smaller than TCXO, which verifies that the OCXO has better clock performance than TCXO. One of the reasons why the high-quality oscillator (e.g., OCXO) is preferred for the application of weak signal processing can be inferred from the figure, as the OCXO can provide tracking capability down to approximately 15 dB-Hz of *C/N*_0_ even for *T* = 20 ms. When the dynamic stress exists, the two oscillators have similar effects on the lower limit, and as the dynamics get stronger, the limits grow because the required gains increase as well.

**Figure 9:**
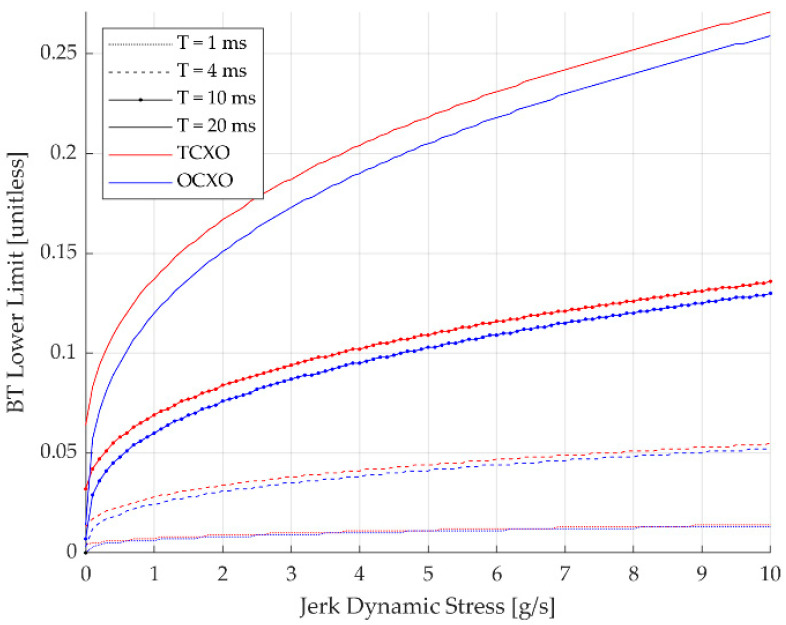
*BT* lower limits of third-order DPLL for jerk dynamic stresses.

The obtained *BT* lower limits of third-order DPLL are arranged in Table 7. Figure 9 shows the *BT* lower limits for various jerk dynamic stresses visually. It can be observed that shorter *T* is beneficial in the dynamic environment since the *T* = 1 ms have nearly the same *BT* limits for all jerk values. Additionally, OCXO has more advantages than TCXO when a longer *T* is used, as the difference between the *BT* limits of the oscillators becomes larger for the longer *T*.

## 5. Implementation and Caveats

**Figure 10:**
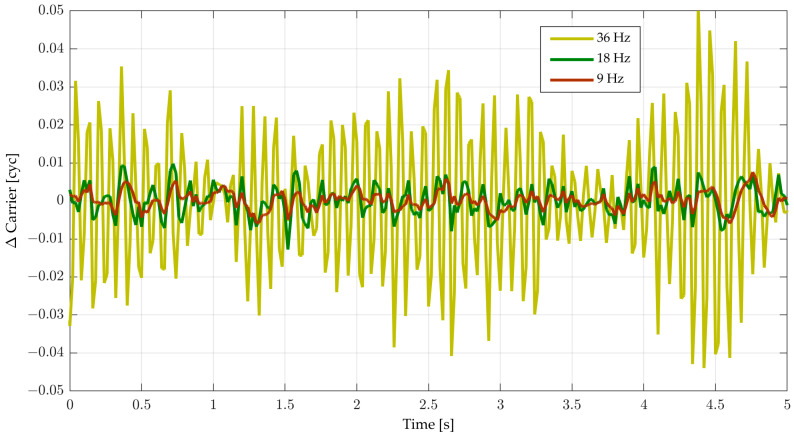
Carrier phase tracking error for *T* = 20 ms, a second-order DPLL with SI/SI numerical integration, *t*_D_ = 0, *C/N*_0_ = 47.7 dB-Hz and various bandwidths.

Focusing on the actual implementation of a second-order DPLL, the step-invariant model for the NCO and loop filter is commonly used. This model is implemented on a GNSS software receiver. For a simulated binary phase shift keying with a chipping rate of 1.023 MHz pilot signal with a constant Doppler shift, the carrier phase tracking error is shown in Figure 10. The largest bandwidth (36 Hz) results in *BT* = 0.72, which is near the stability limit (*BT*_osc_ = 0.75) when *t*_D_ = 0. In this case, oscillations are visible, but carrier tracking remains stable.

At first glance, the implementation of a DPLL seems straightforward; however, two important flaws should be taken care of. Therefore, they are considered and implemented in the receiver to see the effects on the phase tracking performance of the DPLL.

### 5.1. Incorrect Carrier Phase Reference Epoch

The correlator integrates correlation results from *t* to *t* + *T* and dumps it at *t* + *T*, then the phase detector (discriminator) estimates the error between the received true phase and the estimated phase in the NCO at *t* + *T*. The loop filter predicts the next phase and phase rate for *t* + 2*T* and controls the NCO accordingly. However, the phase error obtained at *t* + *T* actually indicates the phase error at *t* + *T*/2. This is because the integrated correlator outputs are the mean values between *t* and *t* + *T* and the resulting phase error is also the mean phase error during the integration period because it is calculated using the mean correlation results. Since the NCO rate is constant over that period and the incoming phase rate is likewise typically assumed to be constant for *T* in the digital loop, the true and estimated phase and the phase error change linearly in that duration. Consequently, the mean phase error reasonably represents the phase error for the midpoint in the integration process (which is at *t* + *T*/2 in this case).

The reference epoch of the estimated phase can be defined differently by each receiver designer. Defining the phase to represent the start point of the integration has an effect such as the insertion of a delay for *T*/2 (half-sample delay). In this work, for the simplicity of the analysis to easily assess the upper and lower limits of *BT*, it is assumed that the carrier phase is defined at the midpoint of the integration interval without any delay, which is an ideal case. This definition is reasonable because the possible Doppler error does not affect the carrier phase discriminator value, and the delay effect on carrier measurement can be compensated later at the measurement extraction module in the baseband process of receivers.

**Figure 11:**
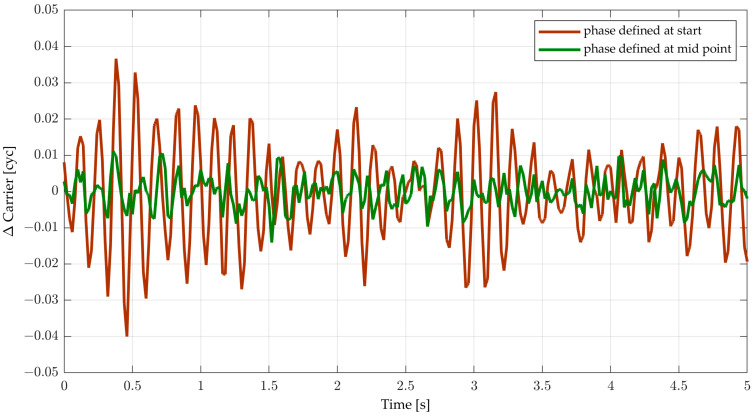
Carrier phase tracking error for *T* = 20 ms, a 20 Hz second-order DPLL with SI/SI numerical integration, *t*_D_ = 0, *C/N*_0_ = 47.7 dB-Hz, and two different carrier phase reference epochs.

On the other hand, a real-world implementation of a DPLL might use a carrier phase value defined at the beginning of the integration interval for convenience. In this case, a Doppler error affects the carrier phase discriminator output (i.e., the Doppler-based phase error is the Doppler error multiplied by *T*/2). If this assumption is used (without accounting for the Doppler error) in combination with the digitization scheme presented here, the tracking stability is degraded. This phenomenon is shown in Figure 11. In fact, the stability drops for a second-order PLL with the SI/SI scheme from *BT* = 0.75 to *BT*~0.4 (determined on an empirical basis) when *t*_D_ = 0.

### 5.2. Computational Delay

**Figure 12:**
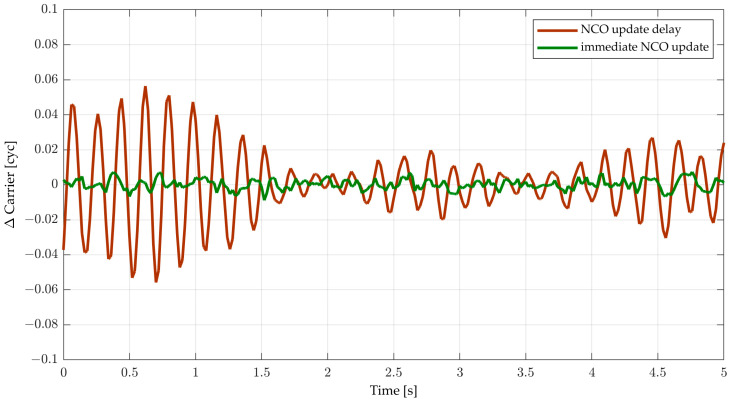
Carrier phase tracking error for *T* = 20 ms, a 13 Hz second-order DPLL with SI/SI numerical integration, *C/N*_0_ = 47.7 dB-Hz, and immediate NCO update (*t*_D_ = 0) and an NCO update delay of 20 ms (*t*_D_ = *T*).

The computation of the discriminator and the loop filter consumes a certain amount of time which is defined as the computational delay as described earlier. Proper buffering of the incoming signal samples can provide the necessary delay to allow an NCO update for the next integration interval. If buffering is not possible, the NCO update must eventually be delayed by one integration period (i.e., *t*_D_ = *T*). The effect of the computational delay is shown in Figure 12 for the second-order DPLL with SI/SI integration, using *BT* = 0.26 which has nearly zero margins when *t*_D_ = *T* (*BT*_osc_ = 0.27). As expected, the result of the NCO update delay case oscillates while the immediate update case does not. One can observe the actual degradation of stability by the computational delay. Furthermore, the result verifies that the obtained *BT* upper limit by root loci in Section 3 is correct.

## 6. Conclusions

In this study, the upper and lower limits of the *BT* for the DPLL were deduced theoretically and presented. For the upper limit, the effects of using digital integration methods on the stability of the DPLLs were investigated using the root loci. Stability problems in sampled-data loops occurred when *BT* was not sufficiently small, such that the sampled-data loops no longer represented their counterparts in the continuous-time domain, which typically occurs in modern digital GNSS receivers. The computational delay inherent in the discriminator and loop filter was considered in the analysis. All the types of DPLLs become conditionally stable when the unit delay is inserted as the computational delay, while the types vary for the order and integration method when zero computational delays are assumed.

The lower limits were obtained by analyzing the DPLL measurement error and the corresponding threshold. The thermal noise, oscillator phase noise using Allan deviation, and dynamic stress error were taken into account to constitute the measurement errors. The *C/N*_0_ threshold was defined as the *C/N*_0_ at the crossing point between the measurement error and threshold. By observing the *C/N*_0_ thresholds for varying *B*, the point that the *C/N*_0_ threshold increases rapidly existed, which is the lower limit. The lower limits are affected by the oscillator quality and dynamic stress because some amount of gain is required for the DPLL to track quickly varying phase and/or phase rate stably.

Issues related to the actual implementation of the DPLL were suggested with the simulation results. As an example, simulation results using second-order DPLL with SI/SI integration were presented. The phase error oscillates as *BT* approaches the deduced upper limits (for both cases with and without the computational delay), which verifies the previous analysis since it behaves as expected. For the numerical analysis of the lower limits, an accurate modeling of the oscillator phase noise on the sampled signal is needed and the dynamic stress, which has a dominant role in determining the lower limits, does not have random characteristics, so it is hard to observe the effect of it using the Monte Carlo simulation. Therefore, such detailed simulation and verification have remained as future works.

## Figures and Tables

**Table 1:** Summary of loop filters.

Loop Order	Loop Filter Transfer Function, *F_i_*(*s*)	Closed-Loop Transfer Function, *H_i_*(*s*)	Typical Filter Values	Sensitive to	Application Examples
First	$F_{1}\left(s\right)=\omega_{0}$	$H_{1}\left(s\right)=\frac{\omega_{0}}{s+\omega_{0}}$	$\omega_{0}=4B$	Velocity stress	Aided code tracking loops
Second	$F_{2}\left(s\right)=\frac{a_{2}\omega_{0}s+\omega_{0}^{2}}{s}$	$H_{2}\left(s\right)=\frac{a_{2}\omega_{0}s+\omega_{0}^{2}}{s^{2}+a_{2}\omega_{0}s+\omega_{0}^{2}}$	$\begin{array}{ccc} \omega_{0} & = & 1.89B\\ a_{2} & = & \sqrt{2} \end{array}$	Acceleration stress	Aided or low-dynamics phase-locked loop, unaided frequency-locked loop
Third	$F_{3}\left(s\right)=\frac{b_{3}\omega_{0}s^{2}+a_{3}\omega_{0}^{2}s+\omega_{0}^{3}}{s^{2}}$	$H_{3}\left(s\right)=\frac{b_{3}\omega_{0}s^{2}+a_{3}\omega_{0}^{2}s+\omega_{0}^{3}}{s^{3}+b_{3}\omega_{0}s^{2}+a_{3}\omega_{0}^{2}s+\omega_{0}^{3}}$	$\begin{array}{ccc} \omega_{0} & = & 1.27B\\ a_{3} & \approx & 1.1\\ b_{3} & \approx & 2.4 \end{array}$	Jerk stress	Unaided phase-locked loop

**Table 2:** Digital equivalences to the analog integrator.

*s*-Domain: Continuous-Time Domain Expression	*z*-Domain	Discrete-Time Domain Expression	Rule	Terminology
$\frac{1}{s}\;↔\;y\left(t\right)=\int x\left(t\right)dt$	$\frac{1}{s}=\frac{T}{z-1}$	$y_{SI}\left(k\right)=y\left(k-1\right)+T\cdot x\left(k-1\right)$	Step-invariant	Forward rule, zero-order holder
	$\frac{1}{s}=\frac{Tz}{z-1}$	$y_{II}\left(k\right)=y\left(k-1\right)+T\cdot x\left(k\right)$	Impulse-invariant	Backward rule, box car
	$\frac{1}{s}=\frac{T}{2}\frac{z+1}{z-1}$	$y_{BL}\left(k\right)=y\left(k-1\right)+\frac{T}{2}\left[x\left(k\right)+x\left(k-1\right)\right]$	Bilinear	Trapezoid, Tustin

**Table 3:** Closed-loop filter coefficients for different digital representations for the integrator in a loop filter and a numerically controlled oscillator (NCO) (zero computational delay assumed).

Closed-Loop Transfer Function: $H\left(s\right)=\frac{N\left(s\right)F\left(s\right)}{1+N\left(s\right)F\left(s\right)}$	*F*(*s*) Transform Method	*N*(*s*) Transform Method		
		Step-Invariant $s\leftarrow \frac{z-1}{T}$	Impulse-Invariant $s\leftarrow \frac{z-1}{Tz}$	$\mathrm{Bilinear}\;s\leftarrow \frac{2}{T}\frac{z-1}{z+1}$
First-order loop $\begin{array}{ccc} H_{1}\left(s\right) & = & \frac{\omega_{0}}{s+\omega_{0}}\\ \omega_{0} & = & 4B \end{array}$	-	$\beta_{0}=T\omega_{0}\;\alpha_{1}=1\;\alpha_{0}=T\omega_{0}-1$	$\beta_{1}=T\omega_{0}\;\beta_{0}=0\;\alpha_{1}=T\omega_{0}+1\;\alpha_{0}=-1$	$\beta_{1}=T\omega_{0}\;\beta_{0}=T\omega_{0}\;\alpha_{1}=T\omega_{0}+2\;\alpha_{0}=T\omega_{0}-2$
Second-order loop $\begin{array}{ccc} H_{2}\left(s\right) & = & \frac{a_{2}\omega_{0}s+\omega_{0}^{2}}{s^{2}+a_{2}\omega_{0}s+\omega_{0}^{2}}\\ \omega_{0} & = & 1.89B\\ a_{2} & = & \sqrt{2} \end{array}$	Step-invariant $s\leftarrow \frac{z-1}{T}$	$\beta_{1}=a_{2}T\omega_{0}\;\beta_{0}=T^{2}\omega_{0}^{2}-a_{2}T\omega_{0}\;\alpha_{2}=1\;\alpha_{1}=a_{2}T\omega_{0}-2\;\alpha_{0}=T^{2}\omega_{0}^{2}-a_{2}T\omega_{0}+1$	$\beta_{2}=a_{2}T\omega_{0}\;\beta_{1}=T^{2}\omega_{0}^{2}-a_{2}T\omega_{0}\;\beta_{0}=0\;\alpha_{2}=a_{2}T\omega_{0}+1\;\alpha_{1}=T^{2}\omega_{0}^{2}-a_{2}T\omega_{0}-2\;\alpha_{0}=1$	$\beta_{2}=a_{2}T\omega_{0}\;\beta_{1}=T^{2}\omega_{0}^{2}\;\beta_{0}=T^{2}\omega_{0}^{2}-a_{2}T\omega_{0}\;\alpha_{2}=a_{2}T\omega_{0}+2\;\alpha_{1}=T^{2}\omega_{0}^{2}-4\;\alpha_{0}=T^{2}\omega_{0}^{2}-a_{2}T\omega_{0}+2$
	Impulse-invariant $s\leftarrow \frac{z-1}{Tz}$	$\beta_{1}=T^{2}\omega_{0}^{2}+a_{2}T\omega_{0}\;\beta_{0}=-a_{2}T\omega_{0}\;\alpha_{2}=1\;\alpha_{1}=T^{2}\omega_{0}^{2}+a_{2}T\omega_{0}-2\;\alpha_{0}=-a_{2}T\omega_{0}+1$	$\beta_{2}=T^{2}\omega_{0}^{2}+a_{2}T\omega_{0}\;\beta_{1}=-a_{2}T\omega_{0}\;\beta_{0}=0\;\alpha_{2}=T^{2}\omega_{0}^{2}+a_{2}T\omega_{0}+1\;\alpha_{1}=-a_{2}T\omega_{0}-2\;\alpha_{0}=1$	$\beta_{2}=T^{2}\omega_{0}^{2}+a_{2}T\omega_{0}\;\beta_{1}=T^{2}\omega_{0}^{2}\;\beta_{0}=-a_{2}T\omega_{0}\;\alpha_{2}=T^{2}\omega_{0}^{2}+a_{2}T\omega_{0}+2\;\alpha_{1}=T^{2}\omega_{0}^{2}-4\;\alpha_{0}=-a_{2}T\omega_{0}+2$
	Bilinear $s\leftarrow \frac{2}{T}\frac{z-1}{z+1}$	$\beta_{1}=T^{2}\omega_{0}^{2}+2a_{2}T\omega_{0}\;\beta_{0}=T^{2}\omega_{0}^{2}-2a_{2}T\omega_{0}\;\alpha_{2}=2\;\alpha_{1}=T^{2}\omega_{0}^{2}+2a_{2}T\omega_{0}-4\;\alpha_{0}=T^{2}\omega_{0}^{2}-2a_{2}T\omega_{0}+2$	$\beta_{2}=T^{2}\omega_{0}^{2}+2a_{2}T\omega_{0}\;\beta_{1}=T^{2}\omega_{0}^{2}-2a_{2}T\omega_{0}\;\beta_{0}=0\;\alpha_{2}=T^{2}\omega_{0}^{2}+2a_{2}T\omega_{0}+2\;\alpha_{1}=T^{2}\omega_{0}^{2}-2a_{2}T\omega_{0}-4\;\alpha_{0}=2$	$\beta_{2}=T^{2}\omega_{0}^{2}+2a_{2}T\omega_{0}\;\beta_{1}=2T^{2}\omega_{0}^{2}\;\beta_{0}=T^{2}\omega_{0}^{2}-2a_{2}T\omega_{0}\;\alpha_{2}=T^{2}\omega_{0}^{2}+2a_{2}T\omega_{0}+4\;\alpha_{1}=2T^{2}\omega_{0}^{2}-8\;\alpha_{0}=T^{2}\omega_{0}^{2}-2a_{2}T\omega_{0}+4$
Third-order loop $\begin{array}{ccc} H_{3}\left(s\right) & = & \frac{b_{3}\omega_{0}s^{2}+a_{3}\omega_{0}^{2}s+\omega_{0}^{3}}{s^{3}+b_{3}\omega_{0}s^{2}+a_{3}\omega_{0}^{2}s+\omega_{0}^{3}}\\ \omega_{0} & = & 1.2B\\ a_{3} & \approx & 1.1\\ b_{3} & \approx & 2.4 \end{array}$	Step-invariant $s\leftarrow \frac{z-1}{T}$	$\beta_{2}=b_{3}T\omega_{0}\;\beta_{1}=a_{3}T^{2}\omega_{0}^{2}-2b_{3}T\omega_{0}\;\beta_{0}=T^{3}\omega_{0}^{3}-a_{3}T^{2}\omega_{0}^{2}+b_{3}T\omega_{0}\;\alpha_{3}=1\;\alpha_{2}=b_{3}T\omega_{0}-3\;\alpha_{1}=a_{3}T^{2}\omega_{0}^{2}-2b_{3}T\omega_{0}+3\;\alpha_{0}=T^{3}\omega_{0}^{3}-a_{3}T^{2}\omega_{0}^{2}+b_{3}T\omega_{0}-1$	$\beta_{3}=b_{3}T\omega_{0}\;\beta_{2}=a_{3}T^{2}\omega_{0}^{2}-2b_{3}T\omega_{0}\;\beta_{1}=T^{3}\omega_{0}^{3}-a_{3}T^{2}\omega_{0}^{2}+b_{3}T\omega_{0}\;\beta_{0}=0\;\alpha_{3}=b_{3}T\omega_{0}+1\;\alpha_{2}=a_{3}T^{2}\omega_{0}^{2}-2b_{3}T\omega_{0}-3\;\alpha_{1}=T^{3}\omega_{0}^{3}-a_{3}T^{2}\omega_{0}^{2}+b_{3}T\omega_{0}+3\;\alpha_{0}=-1$	$\beta_{3}=b_{3}T\omega_{0}\;\beta_{2}=a_{3}T^{2}\omega_{0}^{2}-b_{3}T\omega_{0}\;\beta_{1}=T^{3}\omega_{0}^{3}-b_{3}T\omega_{0}\;\beta_{0}=T^{3}\omega_{0}^{3}-a_{3}T^{2}\omega_{0}^{2}+b_{3}T\omega_{0}\;\alpha_{3}=b_{3}T\omega_{0}+2\;\alpha_{2}=a_{3}T^{2}\omega_{0}^{2}-b_{3}T\omega_{0}-6\;\alpha_{1}=T^{3}\omega_{0}^{3}-b_{3}T\omega_{0}+6\;\alpha_{0}=T^{3}\omega_{0}^{3}-a_{3}T^{2}\omega_{0}^{2}+b_{3}T\omega_{0}-2$
	Impulse-invariant $s\leftarrow \frac{z-1}{Tz}$	$\beta_{2}=T^{3}\omega_{0}^{3}+a_{3}T^{2}\omega_{0}^{2}+b_{3}T\omega_{0}\;\beta_{1}=-a_{3}T^{2}\omega_{0}^{2}-2b_{3}T\omega_{0}\;\beta_{0}=b_{3}T\omega_{0}\;\alpha_{3}=1\;\alpha_{2}=T^{3}\omega_{0}^{3}+a_{3}T^{2}\omega_{0}^{2}+b_{3}T\omega_{0}-3\;\alpha_{1}=-a_{3}T^{2}\omega_{0}^{2}-2b_{3}T\omega_{0}+3\;\alpha_{0}=b_{3}T\omega_{0}-1$	$\beta_{3}=T^{3}\omega_{0}^{3}+a_{3}T^{2}\omega_{0}^{2}+b_{3}T\omega_{0}\;\beta_{2}=-a_{3}T^{2}\omega_{0}^{2}-2b_{3}T\omega_{0}\;\beta_{1}=b_{3}T\omega_{0}\;\beta_{0}=0\;\alpha_{3}=T^{3}\omega_{0}^{3}+a_{3}T^{2}\omega_{0}^{2}+b_{3}T\omega_{0}+1\;\alpha_{2}=-a_{3}T^{2}\omega_{0}^{2}-2b_{3}T\omega_{0}-3\;\alpha_{1}=b_{3}T\omega_{0}+3\;\alpha_{0}=-1$	$\beta_{3}=T^{3}\omega_{0}^{3}+a_{3}T^{2}\omega_{0}^{2}+b_{3}T\omega_{0}\;\beta_{2}=T^{3}\omega_{0}^{3}-b_{3}T\omega_{0}\;\beta_{1}=-a_{3}T^{2}\omega_{0}^{2}-b_{3}T\omega_{0}\;\beta_{0}=b_{3}T\omega_{0}\;\alpha_{3}=T^{3}\omega_{0}^{3}+a_{3}T^{2}\omega_{0}^{2}+b_{3}T\omega_{0}+2\;\alpha_{2}=T^{3}\omega_{0}^{3}-b_{3}T\omega_{0}-6\;\alpha_{1}=-a_{3}T^{2}\omega_{0}^{2}-b_{3}T\omega_{0}+6\;\alpha_{0}=b_{3}T\omega_{0}-2$
	Bilinear $s\leftarrow \frac{2}{T}\frac{z-1}{z+1}$	$\beta_{2}=T^{3}\omega_{0}^{3}+2a_{3}T^{2}\omega_{0}^{2}+4b_{3}T\omega_{0}\;\beta_{1}=2T^{3}\omega_{0}^{3}-8b_{3}T\omega_{0}\;\beta_{0}=T^{3}\omega_{0}^{3}-2a_{3}T^{2}\omega_{0}^{2}+4b_{3}T\omega_{0}\;\alpha_{3}=4\;\alpha_{2}=T^{3}\omega_{0}^{3}+2a_{3}T^{2}\omega_{0}^{2}+4b_{3}T\omega_{0}-12\;\alpha_{1}=2T^{3}\omega_{0}^{3}-8b_{3}T\omega_{0}+12\;\alpha_{0}=T^{3}\omega_{0}^{3}-2a_{3}T^{2}\omega_{0}^{2}+4b_{3}T\omega_{0}-4$	$\beta_{3}=T^{3}\omega_{0}^{3}+2a_{3}T^{2}\omega_{0}^{2}+4b_{3}T\omega_{0}\;\beta_{2}=2T^{3}\omega_{0}^{3}-8b_{3}T\omega_{0}\;\beta_{1}=T^{3}\omega_{0}^{3}-2a_{3}T^{2}\omega_{0}^{2}+4b_{3}T\omega_{0}\;\beta_{0}=0\;\alpha_{3}=T^{3}\omega_{0}^{3}+2a_{3}T^{2}\omega_{0}^{2}+4b_{3}T\omega_{0}+4\;\alpha_{2}=2T^{3}\omega_{0}^{3}-8b_{3}T\omega_{0}-12\;\alpha_{1}=T^{3}\omega_{0}^{3}-2a_{3}T^{2}\omega_{0}^{2}+4b_{3}T\omega_{0}+12\;\alpha_{0}=-4$	$\beta_{3}=T^{3}\omega_{0}^{3}+2a_{3}T^{2}\omega_{0}^{2}+4b_{3}T\omega_{0}\;\beta_{2}=3T^{3}\omega_{0}^{3}+2a_{3}T^{2}\omega_{0}^{2}-4b_{3}T\omega_{0}\;\beta_{1}=3T^{3}\omega_{0}^{3}-2a_{3}T^{2}\omega_{0}^{2}-4b_{3}T\omega_{0}\;\beta_{0}=T^{3}\omega_{0}^{3}-2a_{3}T^{2}\omega_{0}^{2}+4b_{3}T\omega_{0}\;\alpha_{3}=T^{3}\omega_{0}^{3}+2a_{3}T^{2}\omega_{0}^{2}+4b_{3}T\omega_{0}+8\;\alpha_{2}=3T^{3}\omega_{0}^{3}+2a_{3}T^{2}\omega_{0}^{2}-4b_{3}T\omega_{0}-24\;\alpha_{1}=3T^{3}\omega_{0}^{3}-2a_{3}T^{2}\omega_{0}^{2}-4b_{3}T\omega_{0}+24\;\alpha_{0}=T^{3}\omega_{0}^{3}-2a_{3}T^{2}\omega_{0}^{2}+4b_{3}T\omega_{0}-8$

**Table 4:** Three types of DPLL systems regarding stability.

Type	Descriptions	Remarks
A	|*z*| ≤ 1 only when *BT* ≤ *BT*_osc_, otherwise |*z*| > 1	Conditionally stable only when *BT* ≤ *BT*_osc_
B	|*z*| ≤ 1 for all *BT*s, but |*z*| → 1 as *BT* increases	The instability increases as *BT* increases
C	|*z*| < 1 for all *BT*s, and |*z*|~0 as *BT* increases	Unconditionally stable

**Table 5:** Marginal stability conditions of the *BT* for different integration methods in the loop filter and NCO.

Filter Order	NCO, *N*(*z*)	Loop Filter, *F*(*z*)	Zero Computational Delay (*t*_D_ = 0)		Unit Computational Delay (*t*_D_ = *T*)	
			*BT*_osc_ *	Type	*BT*_osc_	Type
First	SI	-	0.51	A	0.26	A
	II		No limit	C	0.51	A
	BL		|*z*|~1 when *BT* >> 1	B	0.51	A
Second	SI	SI	0.75	A	0.27	A
		II	0.55	A	0.25	A
		BL	0.75	A	0.27	A
	II	SI	2.05	A	0.75	A
		II	No limit	C	0.55	A
		BL	|*z*|~1 when *BT* >> 1	B	0.75	A
	BL	SI	1.5	A	0.41	A
		II	|*z*|~1 when *BT* >> 1	B	0.43	A
		BL	|*z*|~1 when *BT* >> 1	B	0.44	A
Third	SI	SI	0.53	A	0.38	A
		II	0.58	A	0.29	A
		BL	0.70	A	0.33	A
	II	SI	0.57	A	0.53	A
		II	No limit	C	0.58	A
		BL	|*z*|~1 when *BT* >> 1	B	0.70	A
	BL	SI	0.53	A	0.51	A
		II	|*z*|~1 when *BT* >> 1	B	0.49	A
		BL	|*z*|~1 when *BT* >> 1	B	0.60	A

* Values for *BT* should be equal to or less than this value for stability.

**Table 6:** Clock parameters for the calculation of the Allan deviation oscillator phase noise.

Oscillator Type	*h*_0_ [s]	*h*_−1_ [-]	*h*_−2_ [1/s]
Temperature-compensated crystal oscillator (TCXO)	1.00 × 10^−21^	1.00 × 10^−20^	2.00 × 10^−20^
Oven-controlled crystal oscillator (OCXO)	2.51 × 10^−26^	2.51 × 10^−23^	2.51 × 10^−22^

**Table 7:** *BT* lower limits of third-order DPLL.

Jerk Stress [g/s]	Oscillator Type	*BT* Lower Limit			
		*T* = 1 ms	*T* = 4 ms	*T* = 10 ms	*T* = 20 ms
0	TCXO	0.004	0.013	0.032	0.064
	OCXO	<0.001	0.003	0.007	0.014
1	TCXO	0.007	0.028	0.069	0.137
	OCXO	0.006	0.024	0.060	0.120
4	TCXO	0.011	0.041	0.102	0.204
	OCXO	0.010	0.038	0.095	0.190
10	TCXO	0.014	0.055	0.136	0.271
	OCXO	0.013	0.052	0.130	0.259

**Figure A1:**
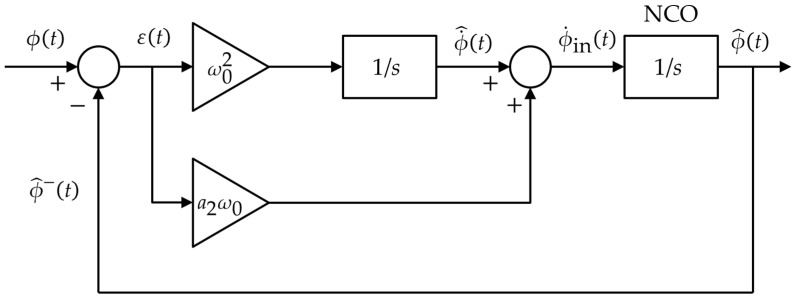
Second-order PLL.

## Data Availability

Not applicable.

## Appendix A

A simple integrator in the continuous-time domain and its corresponding numerical implementations using SI, II, and BL are given by:

(A1) $$y\left(t\right)=\int_{t=0}^{T}x\left(t\right)dt$$

and:

(A2) $$\begin{array}{cc} y_{SI}\left(k\right) & =y\left(k-1\right)+T\cdot x\left(k-1\right)\\ y_{II}\left(k\right) & =y\left(k-1\right)+T\cdot x\left(k\right)\\ y_{BL}\left(k\right) & =y\left(k-1\right)+\frac{T}{2}\left[x\left(k\right)+x\left(k-1\right)\right] \end{array}$$

where *x* and *y* are the input and output of the integrator, respectively, as in Table 2, and the subscripts represent each numerical integration method.

Let us consider a second-order PLL, for example. Figure A1 shows the block diagram of the linearized model of the second-order PLL in the continuous-time domain, where the analog integrator is represented by 1/*s* [2]. In the figure, *ε*(*t*) represents the discriminator output at *t*, which is obtained by subtracting the phase estimate at the previous loop update epoch, ${\hat{\phi }}^{-}\left(t\right)$, from the phase measurement at *t*, $\phi \left(t\right)$. Similarly, the discriminator output is calculated in the discrete-time domain as follows:

(A3) $$\varepsilon \left(k\right)=\phi \left(k\right)-{\hat{\phi }}^{-}\left(k\right)=\phi \left(k\right)-\hat{\phi }\left(k-1\right)$$

The discriminator output is multiplied by the multiplier coefficients, which are based on the natural frequency of the system (i.e., *ω*_0_). The output is then processed as shown in the figure.

The digital implementation of the integrator in *F*(*z*) is obtained by applying (A2) to $\hat{\dot{\phi }}$:

(A4) $$\begin{array}{cc} {\hat{\dot{\phi }}}_{SI}\left(k\right) & =\hat{\dot{\phi }}\left(k-1\right)+T\cdot \omega_{0}^{2}\varepsilon \left(k-1\right)\\ {\hat{\dot{\phi }}}_{II}\left(k\right) & =\hat{\dot{\phi }}\left(k-1\right)+T\cdot \omega_{0}^{2}\varepsilon \left(k\right)\\ {\hat{\dot{\phi }}}_{BL}\left(k\right) & =\hat{\dot{\phi }}\left(k-1\right)+\frac{T}{2}\cdot \omega_{0}^{2}\left[\varepsilon \left(k\right)+\varepsilon \left(k-1\right)\right] \end{array}$$

The control signal of the NCO, which is the estimate of the phase rate, is obtained by the summation of $\hat{\dot{\phi }}\left(k\right)$ and the output from the component with *a*_2_*ω*_0_:

(A5) $${\dot{\phi }}_{in}\left(k\right)=\hat{\dot{\phi }}\left(k\right)+a_{2}\omega_{0}\varepsilon \left(k\right)$$

where *a*_2_ is the filter coefficient of the second-order PLL.

The digital implementation of *N*(*z*) is obtained by applying (A2) and (A5) to $\hat{\phi }$:

(A6) $$\begin{array}{cc} {\hat{\phi }}_{SI}\left(k\right) & =\hat{\phi }\left(k-1\right)+T\cdot {\dot{\phi }}_{in}\left(k-1\right)\\ & =\hat{\phi }\left(k-1\right)+T\cdot \hat{\dot{\phi }}\left(k-1\right)+T\cdot a_{2}\omega_{0}\varepsilon \left(k-1\right)\\ {\hat{\phi }}_{II}\left(k\right) & =\hat{\phi }\left(k-1\right)+T\cdot {\dot{\phi }}_{in}\left(k\right)\\ & =\hat{\phi }\left(k-1\right)+T\cdot \hat{\dot{\phi }}\left(k\right)+T\cdot a_{2}\omega_{0}\varepsilon \left(k\right)\\ {\hat{\phi }}_{BL}\left(k\right) & =\hat{\phi }\left(k-1\right)+\frac{T}{2}\left[{\dot{\phi }}_{in}\left(k\right)+{\dot{\phi }}_{in}\left(k-1\right)\right]\\ & =\hat{\phi }\left(k-1\right)+\frac{T}{2}\left[\hat{\dot{\phi }}\left(k\right)+\hat{\dot{\phi }}\left(k-1\right)\right]+\frac{T}{2}\cdot a_{2}\omega_{0}\left[\varepsilon \left(k\right)+\varepsilon \left(k-1\right)\right] \end{array}$$

Similarly, the digital implementation of *F*(*z*) and *N*(*z*) for the first-/third-order cases can be obtained. Finally, the digital implementation of the overall DPLL for the combinations of the three integration methods is listed in Table A1.

**Table A1:** DPLL implementations for three numerical integration methods.

Filter Order	Type		DPLL Implementations
First	*N*(*z*)	SI	${\hat{\phi }}_{SI}\left(k\right)=\hat{\phi }\left(k-1\right)+T\cdot \omega_{0}\varepsilon \left(k-1\right)$
		II	${\hat{\phi }}_{II}\left(k\right)=\hat{\phi }\left(k-1\right)+T\cdot \omega_{0}\varepsilon \left(k\right)$
		BL	${\hat{\phi }}_{BL}\left(k\right)=\hat{\phi }\left(k-1\right)+\frac{T}{2}\cdot \omega_{0}\left[\varepsilon \left(k\right)+\varepsilon \left(k-1\right)\right]$
Second	*F*(*z*)	SI	${\hat{\dot{\phi }}}_{SI}\left(k\right)=\hat{\dot{\phi }}\left(k-1\right)+T\cdot \omega_{0}^{2}\varepsilon \left(k-1\right)$
		II	${\hat{\dot{\phi }}}_{II}\left(k\right)=\hat{\dot{\phi }}\left(k-1\right)+T\cdot \omega_{0}^{2}\varepsilon \left(k\right)$
		BL	${\hat{\dot{\phi }}}_{BL}\left(k\right)=\hat{\dot{\phi }}\left(k-1\right)+\frac{T}{2}\cdot \omega_{0}^{2}\left[\varepsilon \left(k\right)+\varepsilon \left(k-1\right)\right]$
	*N*(*z*)	SI	${\hat{\phi }}_{SI}\left(k\right)=\hat{\phi }\left(k-1\right)+T\cdot \hat{\dot{\phi }}\left(k-1\right)+T\cdot a_{2}\omega_{0}\varepsilon \left(k-1\right)$
		II	${\hat{\phi }}_{II}\left(k\right)=\hat{\phi }\left(k-1\right)+T\cdot \hat{\dot{\phi }}\left(k\right)+T\cdot a_{2}\omega_{0}\varepsilon \left(k\right)$
		BL	${\hat{\phi }}_{BL}\left(k\right)=\hat{\phi }\left(k-1\right)+\frac{T}{2}\left[\hat{\dot{\phi }}\left(k\right)+\hat{\dot{\phi }}\left(k-1\right)\right]+\frac{T}{2}\cdot a_{2}\omega_{0}\left[\varepsilon \left(k\right)+\varepsilon \left(k-1\right)\right]$
Third	*F*(*z*)	SI	${\hat{\ddot{\phi }}}_{SI}\left(k\right)=\hat{\ddot{\phi }}\left(k-1\right)+T\cdot \omega_{0}^{3}\varepsilon \left(k-1\right)\;{\hat{\dot{\phi }}}_{SI}\left(k\right)=\hat{\dot{\phi }}\left(k-1\right)+T\cdot {\hat{\ddot{\phi }}}_{SI}\left(k-1\right)+T\cdot a_{3}\omega_{0}^{2}\varepsilon \left(k-1\right)$
		II	${\hat{\ddot{\phi }}}_{II}\left(k\right)=\hat{\ddot{\phi }}\left(k-1\right)+T\cdot \omega_{0}^{3}\varepsilon \left(k\right)\;{\hat{\dot{\phi }}}_{II}\left(k\right)=\hat{\dot{\phi }}\left(k-1\right)+T\cdot {\hat{\ddot{\phi }}}_{II}\left(k\right)+T\cdot a_{3}\omega_{0}^{2}\varepsilon \left(k\right)$
		BL	${\hat{\ddot{\phi }}}_{BL}\left(k\right)=\hat{\ddot{\phi }}\left(k-1\right)+\frac{T}{2}\cdot \omega_{0}^{3}\left[\varepsilon \left(k\right)+\varepsilon \left(k-1\right)\right]\;{\hat{\dot{\phi }}}_{BL}\left(k\right)=\hat{\dot{\phi }}\left(k-1\right)+\frac{T}{2}\left[{\hat{\ddot{\phi }}}_{BL}\left(k\right)+{\hat{\ddot{\phi }}}_{BL}\left(k-1\right)\right]+\frac{T}{2}\cdot a_{3}\omega_{0}^{2}\left[\varepsilon \left(k\right)+\varepsilon \left(k-1\right)\right]$
	*N*(*z*)	SI	${\hat{\phi }}_{SI}\left(k\right)=\hat{\phi }\left(k-1\right)+T\cdot \hat{\dot{\phi }}\left(k-1\right)+T\cdot b_{3}\omega_{0}\varepsilon \left(k-1\right)$
		II	${\hat{\phi }}_{II}\left(k\right)=\hat{\phi }\left(k-1\right)+T\cdot \hat{\dot{\phi }}\left(k\right)+T\cdot b_{3}\omega_{0}\varepsilon \left(k\right)$
		BL	${\hat{\phi }}_{BL}\left(k\right)=\hat{\phi }\left(k-1\right)+\frac{T}{2}\left[\hat{\dot{\phi }}\left(k\right)+\hat{\dot{\phi }}\left(k-1\right)\right]+\frac{T}{2}\cdot b_{3}\omega_{0}\left[\varepsilon \left(k\right)+\varepsilon \left(k-1\right)\right]$

*a_i_*, *b_i_*: filter coefficients for the *i*-th order PLL.
